# MicroRNA‐98 regulates osteogenic differentiation of human bone mesenchymal stromal cells by targeting BMP2

**DOI:** 10.1111/jcmm.12961

**Published:** 2016-11-18

**Authors:** Guo‐Ping Zhang, Jing Zhang, Chao‐Hua Zhu, Lei Lin, Jian Wang, Hai‐Jing Zhang, Jun Li, Xiao‐Guang Yu, Zhen‐Shuan Zhao, Wei Dong, Guo‐Bin Liu

**Affiliations:** ^1^ Department of OrthopedicsThe First Hospital of Hebei Medical UniversityShijiazhuangChina; ^2^ Medical Physics Department of Basic Medical College of Hebei Medical UniversityShijiazhuangChina

**Keywords:** microRNA, microRNA‐98, bone morphogenetic protein‐2, human bone mesenchymal stromal cells, osteogenic differentiation, signal pathway, transcription, translation

## Abstract

To study the effects of microRNA‐98 (miR‐98) on human bone mesenchymal stromal cells (hBMSCs). The patients undergoing hip arthroplasty were selected by inclusion/exclusion criteria for this study. The extracted hBMSCs were detected of osteogenic differentiation by alizarin red S staining, and of cell phenotype by flow cytometry. Bioinformatics, dual luciferase report, western blotting, RT‐PCR and immunoblotting were used in our study. The hBMSCs were divided into miR‐98 mimics, miR‐98 negative control (NC), miR‐98 inhibitors, Mock and miR‐98 inhibitors + siBMP2 groups. Human bone mesenchymal stromal cells were extracted and purified *in vitro* and had specific cytological morphology, surface markers and abilities of self‐renewal and differentiation. Compared with the NC group and Mock group, the miR‐98 mimics group showed increased miR‐98 level while the miR‐98 inhibitors group decreased miR‐98 level (both *P* < 0.01). Dual luciferase reporter showed BMP2 was the target gene of miR‐98. The levels of mRNA and protein expression of BMP2, protein expression of RUNX2, alkaline phosphatase activity and osteocalcin content significantly decreased in the miR‐98 mimics group while increased in the miR‐98 inhibitors group and showed no changes in the NC group and Mock group (all *P* < 0.05). The miR‐98 mimics group showed obviously declined stained red particles and the miR‐98 inhibitors group showed opposite result. After lowering the expression of miR‐98, osteogenic differentiation ability of hBMSCs rose, which was weakened by the transfection with siBMP2. miR‐98 may regulate osteogenic differentiation of hBMSCs by targeting BMP2.

## Introduction

Human bone mesenchymal stromal cells (also known as mesenchymal stem cells, bone marrow stromal cells or multipotent mesenchymal stromal cells) are a group of clonogenic cells in the bone marrow stroma and is capable of self‐renewal and multilineage differentiation into such cell types as mesoderm‐type cells, including osteoblast, adipocyte and chondrocytes, and epithelial cells 1, 2, 3. Mesenchymal progenitor cells go through a multi‐stage differentiation process in skeletal development including proliferation and formation of bone and cartilage cells, which is tightly controlled by multiple levels of regulatory systems 4. According to Papaioannou *et al*., extracellular signalling molecules such as miRNA can trigger bone cells to proliferate and differentiate, which ultimately regulates gene expression and monitor cellular functions 4. Osteogenesis, a highly coordinated process, involves transcription factors such as BMP2, RUNX2 and OSTERIX, and drives stem cells to fully differentiated osteocytes 5, 6, 7, 8. Osteogenesis is promoted through several signalling pathways, including WNT/β‐catenin, BMP, JAK/STAT and MAPK 9, 10, 11.

MicroRNA (miRNA) has been reported to play significant roles throughout stages of bone formation, indicating that miRNAs might possibly become novel therapeutic targets for skeletal related diseases 4. Several miRNAs were demonstrated to get involved in osteogenic differentiation modulation: miR‐125b negatively regulated osteogenic differentiation by targeting ErbB2, VDR and Osterix; miR‐133 and miR‐135 inhibited differentiation of mouse osteoprogenitors, respectively, by targeting RUNX2 and recognizing SMAD5; miR‐26a and miR‐29b promoted osteogenic differentiation of human adipose tissue‐derived stem cells, and positively regulated mouse osteoblast differentiation 12, 13, 14, 15, 16. Besides, a wide range of other miRNAs have links to osteogenesis, to name a few, miR‐9, ‐17, ‐27, ‐30, ‐96, ‐106 and so on 14. Lethal‐7/miR‐98 family, one of the earliest identified mammalian miRNAs, consists of 12 members, let‐7‐a1, a2, a3, b, c, d, e, f1, f2, g, i and miR‐98, which are located on eight different chromosomes 17, 18, 19, 20. Let‐7, expressed in mammalian embryonic development, plays an evolutionarily conservative role from Caenorhabditis elegans to Drosophila to mammals 21, 22. Identified targets of let‐7 include cell cycle regulators, promoters of growth and multiple early embryonic genes 23, 24, 25, 26, 27. Members of the let‐7 family are reported to inhibit cell growth responses and they are recognized as tumour suppressors 28. Let‐7/miR‐98 regulate Fas/Fas‐mediated apoptosis 29; miR‐98 regulates expression of tumour suppressor gene FUS1 30; miR‐98 could inhibit tumour angiogenesis and invasion by targeting activin receptor‐like kinase‐4/matrix metalloproteinase‐11 31; let‐7/miR‐98 might be involved in regulating progesterone receptor membrane component 1 expression in ovarian cancer cells 32. Although no direct research on miR‐98 regulating osteogenic differentiation was found, its effect on cell growth may also have links with skeletal cells; therefore, we carried out the present study based on the hypothesis that miR‐98 may participate in the mechanism of osteogenic differentiation of hBMSCs.

## Materials and methods

### Study objects

Forty‐eight patients included in our study were those hospitalized to undergo hip arthroplasty at the First Hospital of Hebei Medical University from April 2014 to April 2015. Among them, 26 were males and 22 were females, with an average age of 59.1 ± 6.2 years. Inclusion criteria: (*i*) accessible to complete clinical data and imaging data (including hip X‐rays, CT or nuclear magnetic resonance) before surgery; (*ii*) diagnosed with simple hip osteoarthritis or osteoarthritis and traumatic arthritis secondary to congenital hip dysplasia by history checking, physical and image examination (no previous history of long‐term large dose hormone consumption, alcohol abuse, or other autoimmune disease). Exclusion criteria: (*i*) patients with femoral head necrosis (induced by alcohol or hormone); (*ii*) patients with hip joint disease because of autoimmune diseases (rheumatoid arthritis, ankylosing spondylitis, *etc*.); (*iii*) senile osteoporosis fracture patients; (*iv*) other (hemophilia hip joint disease, tuberculosis, suppurative infection, tumours around hip joint, *etc*.). The scheme was approved by the Ethics Committee of the First Hospital of Hebei Medical University. All patients in this study were signed informed consents.

### Isolation of hBMSCs

All patients received artificial hip arthroplasty using aseptic bone marrow puncture needles. Bone marrow (10 ml) from femoral head was extracted during operation and quickly transferred *in vitro* containing medium and anti‐freezing heparin (4000 U/ml; Nanjing Nanda Geotechnical Engineering Co., Ltd., Nanjing, China). After it was evenly mixed, the bone marrow was diluted with equivalent PBS (Nanjing SenBeiJia Biological Technology Co., Ltd, Nanjing, China), followed by an even mixing, rest for 30 sec. and discarding of sediment. The centrifuge tube (20 ml) was added with appropriate amount of lymphocyte separation medium (Tianjin Hao Yang Biological Manufacture, Tianjin, China) and slow drops of diluted bone marrow specimens along the surface layer. After a horizontal centrifugation (134 g × 20 min.), there were white clouds in a narrow layer of mononuclear cells at the upper middle interface, which was carefully drawn and washed with 10 ml D‐Hank'S liquid (Beijing Huamaike Biological Technology Co., Ltd, Beijing, China). The cells were collected after a 48 g × 6 min. centrifugation. All experiments were performed by triplicates.

### Inoculation and culture of hBMSCs

The isolated single cells (of density 2 × 10^6^/ml) were cultured in an inoculator at 37°C with 5% CO_2_ and 95% humidity, which contained 58% DMEM/F12 (Qingdao Jie Shi Kang Biotechnology Co., Ltd, Qingdao, China), 40% MCDB‐201 (Qingdao Jie Shi Kang Biotechnology Co., Ltd), 2% foetal bovine serum (FBS; Tianjin Blood Institute, Tianjin, China), 10 ng/ml epidermal growth factor (EGF; Gibco‐Invitrogen Corporation, Carls‐bad, CA), 10 ng/ml platelet‐derived growth factor (PDGF; Sigma Chemical Company, USA), 1× insulin‐transferrin‐selenium (Gibco‐Invitrogen Corporation), 1× linoleic acid‐bovine serum albumin (Gibco‐Invitrogen Corporation), 50 μM β mercaptoethanol (Gibco‐Invitrogen Corporation), 2 ml glutamine (Gibco‐Invitrogen Corporation), 100 μg/ml penicillin (North China Pharmaceutical Factory, China) and 100 U/ml streptomycin sulphate (North China Pharmaceutical Factory, Shijiazhuang, Hebei, China). The medium was changed 2 days later and the non‐adherent cells were discarded. Half volume of the medium was then changed every 3 days until the cell density reached 70–80%, then the cells were digested with 0.25% trypsin −0.01% ethylene diamine tetraacetic acid (Gibco‐Invitrogen Corporation) and passaged in the ratio of 1:3. All experiments were performed by triplicates. The experimental hBMSCs were the third generation of cells; the cells temporarily not available were reserved in a −80°C refrigerator for further use.

### Differentiation and identification of hBMSCs into osteoblasts

The hBMSCs (of density 2 × 10^4^/cm^2^) were inoculated in a 24‐well plate overnight. When the cell density reached 70–80%, the medium was replaced by osteoinductive culture fluid. The H‐DMEM culture medium (Gibco‐Invitrogen Corporation) was added with 10% FBS (Sigma‐Aldrich Company, St. Louis, MO, USA), 10 nM dexamethasone (Sigma‐Aldrich Company), 0.2 mM ascorbic acid (Sigma‐Aldrich Company) and 10 mM 3‐Sodium aluminate (Sigma‐Aldrich Company) for further culture. Half volume of the medium was then changed every 2 days for 21 days, alizarin red S staining was performed for calcification detection. All experiments were performed by triplicates.

### Alizarin red S staining

The cultured cells were washed 2 times with PBS, fixed for 10 min. in 95% ethanol, rinsed 3 times by double distilled water, followed by staining for 30 min. at 37°C with 0.1% alizarin red‐Tris‐HCL (pH 8.3) (Sigma‐Aldrich Company), washed with distilled water, dried and mounted. The mineralization ability of the cells in induced group and in un‐induced group was detected after 21 days of induction of cell differentiation. The positive result was found with the calcified nodules stained orange red in the extracellular matrix. The Photoshop Image analysis program was used to count the calcified nodules, and then calculated the percentage (%) of the positive calcium nodules in the whole image. All experiments were performed by triplicates, and the average was chosen as the final result.

### Identification of cell surface molecular markers

The hBMSCs in logarithmic growth period were digested with 0.25% trypsin, centrifuged at 134 g, washed twice by PBS, and followed by a 34 g × 5 min. centrifugation at room temperature. The cells were counted for around 1 × 10^6^/ml. Each tube was added with 20 μl fluorescent labelled anti‐human monoclonal antibody CD73‐APC (ab81720; Abcam, Cambridge, UK), CD90‐FITC (ab23894; Abcam), CD105‐PE (ab2529; Abcam), CD14 (ab182032; Abcam)/CD20 (ab8237; Abcam)/CD34 (ab81289; Abcam)/CD45PRer‐CP (ab10559; Abcam) and NC anti‐human IgG l‐FITC (ab150113; Abcam) of the same type and then 100 μl cell suspension, followed by fully mixing, reaction for 15 min. at room temperature in dark, PBS washing, centrifugation for 5 min. (at room temperature, 134 g). After discarding the supernatant, each tube was added with 500 μl PBS and detected with a flow cytometry (FACS Calibur; Becton‐Dickinson, San Diego, CA, USA). Flowjo software was adopted to analyse data. All experiments were performed by triplicates.

### Design and synthesis of BMP2‐siRNA

According to mRNA sequence of human BMP2 gene (NM_001200), siRNA (sense: 5′‐GCAACAGCCAACUCGAAAU dTdT‐3′; Anti‐sense: 5′‐AUUUCGAGUUGGCUGUUGC dTdT‐3′) was designed using a siRNA design software (Invitrogen Inc., Carlsbad, CA, USA). The target sequence corresponding to the siRNA sequence was 19 bases and the G/C ratio was close to 50%. A homology comparison verified that the siRNA had no common sequence with other genes. The sense sequence of siRNA was added by two TT prominent ends in the 3′ end. The chemical synthesis and modification were performed by Guangzhou RiboBio Co., Ltd. (Guangzhou, China).

### Cell transfection

The study included five groups: (*i*) miR‐98 mimics group (transfected synthetic miR‐98 analogues, which were purchased from Shanghai Zimmer Pharmaceutical Company, Shanghai, China, the same hereinafter); (*ii*) miR‐98‐NC group (transfected meaningless sequence, abbreviated as miR‐98‐NC group); (*iii*) miR‐98 inhibitors (transfected miR‐98 inhibitor); (*iv*) mock group (blank control, transfected no sequence); (*v*) miR‐98 inhibitors + siBMP2 group; and (*vi*) siBMP2 group. After inoculation, the cells were cultured for 24 hrs and when the adherent cells reached 30–50%, transfection was performed according to the lipofectamineTM2000 transfection reagent specification. Six hours later, the transfected cells were cultured in replaced culture solution for further use. Transfection of miRNA‐98 mimics is explained as the example. The transfection mixture was prepared with 250 μl OPTI‐MEM medium (Gibco‐Invitrogen Corporation), supplemented with 5 μl miRNA‐98 mimics and 5 μl LipofectamineTM2000 transfection reagent (Invitrogen Inc.) independently by an RNA enzyme free gun head. The mixture was evenly mixed, rested for 20 min. at room temperature, added to the 6‐well plate, followed by an even mixing with the original culture solution and further culture for subsequent experiments. All experiments were performed by triplicates.

### Dual luciferase reporter assay

It was predicted by bioinformatics software that there was a good base complementary relationship between miR‐98 and BMP2, *i.e*., binding sites existed between miR‐98 and the 3′‐untranslated regions (UTR) of BMP2. The 3′UTR of BMP2 promoter sequence, containing miR‐98 binding sites, was synthetized and inserted at the corresponding restriction enzyme cutting site to construct wild‐type (WT) plasmid of the 3′UTR of BMP2. And on the basis of this plasmid, the miR‐98 binding site (GGAUGGAG) was mutated, and constructed the mutant (MT) plasmid of the 3′UTR of BMP2. The hBMSCs were inoculated in a 96‐well plate (about 4 × 10^3^/well) and divided into four groups: miR‐98 mimics + BMP2‐WT group (transfected WT of BMP2 plus miR‐98 mimics), miR‐98 mimics + BMP2‐MT group (transfected MT type of BMP2 plus miR‐98 mimics), miR‐98 NC + BMP‐WT group (transfected WT + NC), and miR‐98 NC + BMP2‐MT group (transfected MT + NC). Six hours later, the medium was replaced; 72 hrs after transfection, hBMSCs were collected and detected for fluorescence signal and sea kidney fluorescence signal in accordance with the dual luciferase reporter gene assay kit. All experiments were performed by triplicates and the average data was adopted as the final result.

### Real‐time quantitative PCR

The total RNA of each group of transfected cells was extracted using miRNeasy Mini Kit (Qiagen Company, Hilden, Germany). An RNA sample (5 μl) was diluted 20 times by RNA enzyme free ultra pure water. The absorption value was detected at 260 and 280 nm points in a UV spectrophotometric meter, with the OD260/OD280 ratio of 1.7–2.1, indicating a high purity of RNA and thus satisfying the need of the experiment. RT reaction was performed in the PCR amplification instrument for synthesis of cDNA template. An ABI7500 quantitative PCR instrument was applied for the RT‐qPCR experiment, with a cDNA template (5.0 μl), upstream and downstream primers (each 0.5 μl), 10× buffer (25 μl), 2× SYBR Green q PCR Super M'iX (10.0 μl) and ddH_2_O (4 μl). The reaction condition was: pre‐denaturation, 94°C for 2 min. and 94°C for 30 sec.; annealing, 53°C for 30 sec.; extension, 72°C for 5 min., which were cycled for 40 times. The primer sequences were shown in Table 1. The lowest points of the logarithmic amplification curves were manually selected as the thresholds. The Ct (threshold cycle) values for each reaction tube were then obtained. The data were analyzed using 2^−ΔΔCt^ method. The relation of target genes expression in the experimental group and the control group were represented as 2^−ΔΔCt^, in which ΔΔCt = [Ct (target gene) − Ct (internal reference gene)] of the experimental group − [Ct (target gene) − Ct (internal reference gene)] of the control group. The experiment was repeated 3 times.

**Table 1:** Primer sequence

Gene	Primer sequence
miR‐98	F: 5′‐GGGACTGGACTTGGAGTCA‐3′
	R: 5′‐GTGCGTGTCGTGGAGTCG‐3′
BMP2	F: 5′‐AACCTGCAACAGCCAACT‐3′
	R: 5′‐GGAGCCACAATCCAGTCAT‐3′
GAPDH	F: 5′‐ACACCATGGGGAAGGTGAAG‐3′
	R: 5′‐AAGGGGTCATTGATGGCAAC‐3′

F: forward; R: reverse.

### Western blotting

Total protein in each transfection group was extracted to prepare protein samples, followed by SDS‐PAGE electrophoresis to separate the samples containing the target protein, which were then transferred onto polyvinylidene fluoride (PVDF) membrane surface *in situ* electrophoresis. After the transmembrane, the PVDF membrane was closed in sealing fluid, and then evenly mixed with diluted RUNX2 antibody (1:200, ab54868; Abcam). This was followed by incubation at 4°C overnight, then the fluid washed 3 times using 1× TBST for 10 min., and incubation for 1 hr after adding with secondary antibody (1:5000, ab6789; Abcam) at room temperature. Working fluid of enhanced chemiluminescent (ECL) solution was prepared and used for 3–5 min. of contact reaction with the positive side of PVDF membrane. After draining the ECL liquid, the PVDF membrane was developed in a darkroom and taken pictures by the Image Master VDS image analysis system. The samples in each group were detected three times, with β‐actin (1:1000, ab194952; Abcam) as an internal reference. All experiments were performed by triplicates and the average data was regarded as the final result.

### Detection of alkaline phosphatase activity

After 72 hrs of cell culture, the alkaline phosphatase (ALP) activity was detected by an ALP activity assay kit (Nanjing Jiancheng Bioengineering Institute, Nanjing, China). According to the manual, the procedures were as followed: the cultured cells were deprived of the medium, washed three times with PBS, lysed by lysis buffer, followed by ultrosonic cell disrupting for release of cell protein and centrifugation for 15 min. (7553 × g, at 4°C); the supernatant was taken out, added with buffer, incubated for 30 min. at 37°C; NaOH was added to cease the reaction, and then the absorption value at 405 nm was determined by the spectrophotometer; the ALP activity was calculated with p2 nitrobenzene as standard value, in which 1 unit represented the activity of 1 nM nitrophenol produced in 30 min.; bisinchoninic acid (BCA) method was used to determine the total intracellular protein, and the ALP activity was corrected. All experiments were performed by triplicates and the average data was regarded as the final result.

### Determination of osteocalcin content

After 72 hrs of cell culture, the supernatant was collected and the osteocalcin (OC) content in it was determined by RIA kit (Sorin Group, Saluggia, Italy); each reagent was added according to the instruction manual. The OC content was modified according to the total protein content, which was determined using BCA method. All experiments were performed by triplicates and the average data was regarded as the final result.

### Statistic analysis

The SPSS 21.0 (IBM‐SPSS Inc., Chicago, IL, USA) software was used for statistical analysis. All data was represented as mean ± S.D., and validated by *t*‐test if complied with the normal distribution and if not by the nonparametric Wilcoxon Rank‐Sum test. ANOVA was performed for the comparisons among groups and *t*‐test for the comparison between two groups. A *P* value less than 0.05 indicated statistically significant.

## Results

### Morphological observation of hBMSCs

**Figure 1:**
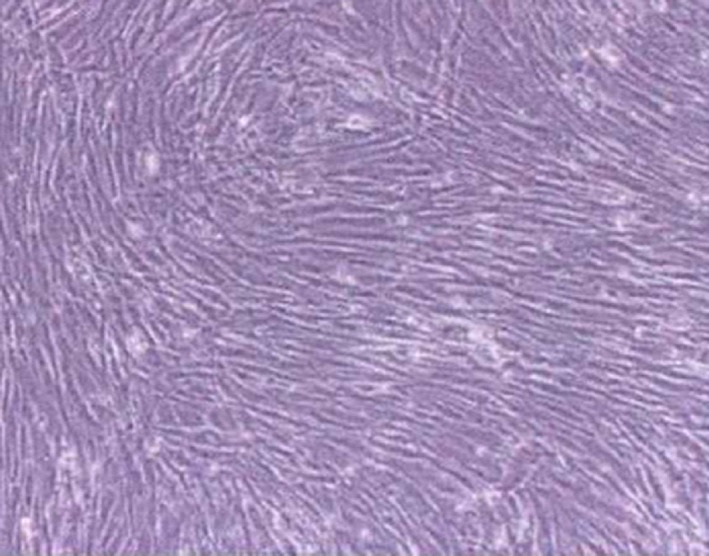
Morphology of hBMSCs (light microscope, ×100). hBMSCs: human bone mesenchymal stromal cells.

The cells were round separated by lymphocyte separation liquid. After 72 hrs of inoculation, half volume of solution was replaced and single spindle shaped adherent cells and a small quality of red blood cells was seen. With prolongation of culture time, adherent cells increased rapidly and turned into long fusiform with a few protuberances, growing as typical fibroblasts. The cells then grew rapidly and formed clones. Approximately 8–12 days later, the cells were 70–80% grown. After *in vitro* amplification, including digestion by pancreatic proteases, inoculation and culture, the cells were purified well and in good growth, retaining their original state (Fig. 1).

### Osteogenic differentiation of hBMSCs

**Figure 2:**
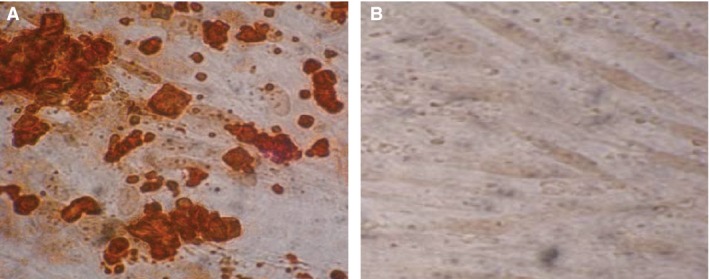
Osteogenic differentiation of hBMSCs. hBMSCs: human bone mesenchymal stromal cells. (**A**) Alizarin red S staining after 21 days of osteogenic induction of hBMSCs; (**B**) Alizarin red S staining after 21 days without osteogenic induction of hBMSCs. Light microscope, ×400.

After osteogenic induction in osteogenic culture system, hBMSCs grew from long spindle shape into polygon or triangle in morphology, with growing body, abundant cytoplasm and multiple thin and short protuberances surrounding cells and large and clear nucleus. Nodular calcium deposits were found in round or oval cells and significantly increased 21 days later. Alizarin red S staining was positive. Under the microscope, the calcified nodules showed different orange red clumps, with the nodular centre deep in colour (Fig. 2A); and no morphological changes occurred in the un‐induced hBMSCs, which were spindle‐shaped, densely distributed, closely packed and grew in single direction and in colonies without calcium nodules (Fig. 2B).

### Surface markers of hBMSCs

**Figure 3:**
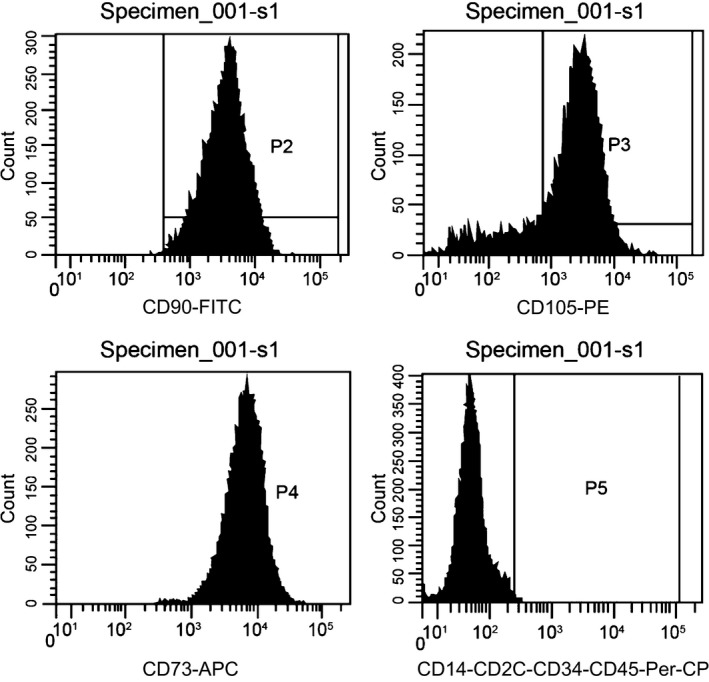
Surface markers detection of hBMSCs. hBMSCs: human bone mesenchymal stromal cells.

At present, no specific markers gained a consensus for the identification of hBMSCs. According to the International Society for Cellular Therapy 33, hBMSCs should have the following characteristics: (*i*) they can grow adherently to a plastic bottle surface when cultured *in vitro*; (*ii*) mesenchymal associated antigens, CD73, CD90 and CD105 are positive, with no expression of hematopoietic stem cell related antigens, CD14, CD20, CD34, CD45; (*iii*) they can differentiate into osteoblasts, steatoblasts and chondroblasts *in vitro* culture, which should be validated at least by staining. Therefore, a flow cytometry was used to detect the cell surface markers and found that hBMSCs highly expressed CD90, CD73 and CD105, with expression rate of (99.02 ± 0.51)%, (99.48 ± 0.32)% and (99.25 ± 0.22)%, respectively, but only a few hematopoietic stem cell related antigens, namely CD14/CD20/CD34/CD45 with expression rate of (0.19 ± 0.04)%, (0.15 ± 0.03)%, (0.18 ± 0.03)% and (0.21 ± 0.05)%, respectively (Fig. 3).

### Target relationship verification of miR‐98 and BMP2

**Figure 4:**
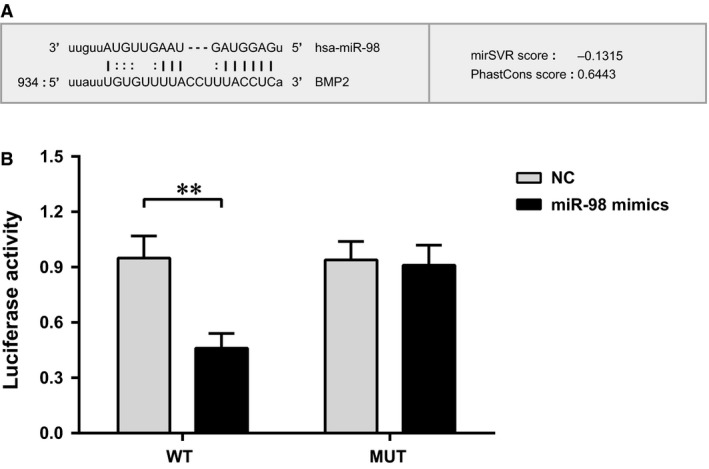
Relative activity of luciferase commonly transfected with BMP2 3′UTR and miR‐98 mimics. (**A**) Base sequence pair graph between miR‐98 and BMP2 3′UTR; (**B**) relative activity of luciferase commonly transfected with BMP2 3′UTR and miR‐98 mimics compared with no transfection; ** represented the *P*‐value less than 0.01. NC: negative control.

Online miRNAs target gene prediction tool miRNA (http://www.microrna.org/) was used to predict the target gene of miR‐98. We found that BMP2 was one of the target genes of miR‐98 and the binding region of BMP2 gene 3′UTR and miR‐98 was highly conserved in mammals (Fig. 4A). As the results of luciferase reporter assay shown, after a co‐transfection by BMP2 3′UTR‐WT and miR‐98 mimic the relative activity of luciferase was significantly decreased (*P* < 0.01), while there were no changes in a co‐transfection by BMP2 3′UTR‐MT and miR‐98 mimic (Fig. 4B). The results were consistent with the prediction of bioinformatics, which further confirmed that miR‐98 was able to combine with the base at the seed region of mRNA 3′UTR of BMP2, suggesting that miR‐98 and BMP2 had targeted relationship.

### Expressions of miR‐98 and BMP2 in hBMSCs

**Figure 5:**
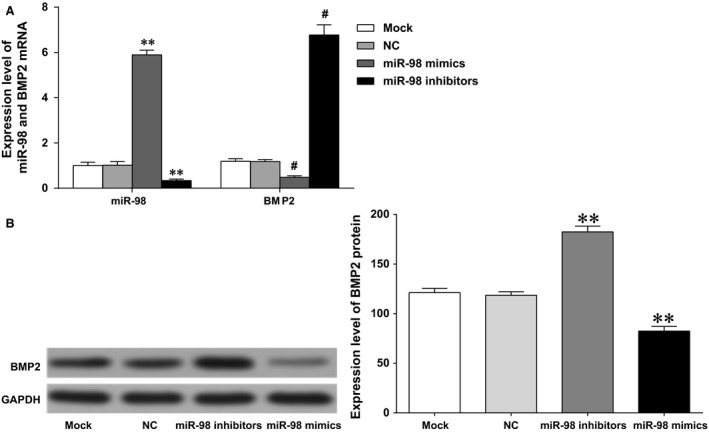
Expressions of miR‐98 and BMP2 in each group. (**A**) qPCR was performed to determine the expressions of miR‐98 and BMP2 in each group; (**B**) western blotting was performed to determine the protein expression of BMP2 in each group; ** and # represented the *P*‐value less than 0.01 compared with the Mock group and the NC group, respectively. NC: negative control.

The level of miR‐98 significantly increased in the miR‐98 mimics group compared to that in the NC group and the Mock group, while significantly decreased in the miR‐98 inhibitors group (both *P* < 0.01), and showed no significant difference in the NC group and the Mock group (both *P* > 0.05). The mRNA and protein levels of BMP2 declined significantly in the miR‐98 mimics group than that in the NC group and the Mock group, increased significantly in the miR‐98 inhibitors group (both *P* < 0.05), and showed no significant difference in the NC group and the Mock group (both *P* > 0.05) (Fig. 5).

### Effect of miR‐98 targeting BMP2 on the osteogenic differentiation ability in hBMSCs

**Figure 6:**
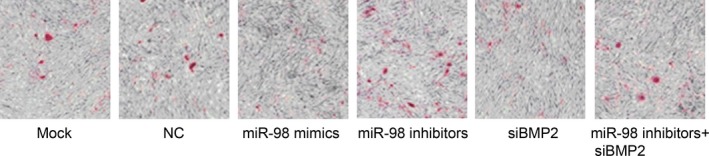
Calcium nodules in each group of cells after 21 days of osteogenic induction. NC: negative control. Light microscope, ×100.

Alizarin red S staining was applied for identification of calcium nodules in osteogenic differentiation, with existing calcium ions forming chelate complexes in tissue cells. Since BMP‐2 played an important role in the early stage of osteogenic differentiation, we added dexamethasone in the culture medium. Stained red and orange sections were calcified nodules in the cells. The calcification ability of the cells was detected after 21 days of cell differentiation. Compared with the NC group (4.9 ± 1.6)% and Mock group (5.0 ± 1.6)%, the stained red particles were significantly reduced in the miR‐98 mimics group (2.1 ± 1.1)%, while increased in the miR‐98 inhibitors group (9.4 ± 1.5)%. To further understand the effect of BMP2 signalling pathway activation on miR‐98 inhibiting osteogenic differentiation of hBMSCs, we knocked down the expression of miR‐98 and simultaneously added siBMP2 processing to inhibit the promotion of miR‐98 on inducing BMP2 activation, after which the osteogenic differentiation ability change was observed. Low‐expression of miR‐98 contributed to an increased osteogenic differentiation ability of the cells, while the transfection of siBMP2 could reverse the osteogenic differentiation of the cells induced by low‐expressed miR‐98. Compared with the miR‐98 inhibitors + siBMP2 group (5.1 ± 2.0)%, the stained red particles were significantly reduced in the siBMP2 group (2.4 ± 1.0)% (Fig. 6). These results indicated that the inhibitory effect of miR‐98 on the osteogenic differentiation of hBMSCs was mainly caused by its inhibition of BMP2 signalling pathway.

### Effect of miR‐98 targeting BMP2 on the levels of osteogenic‐related markers in hBMSCs

**Figure 7:**
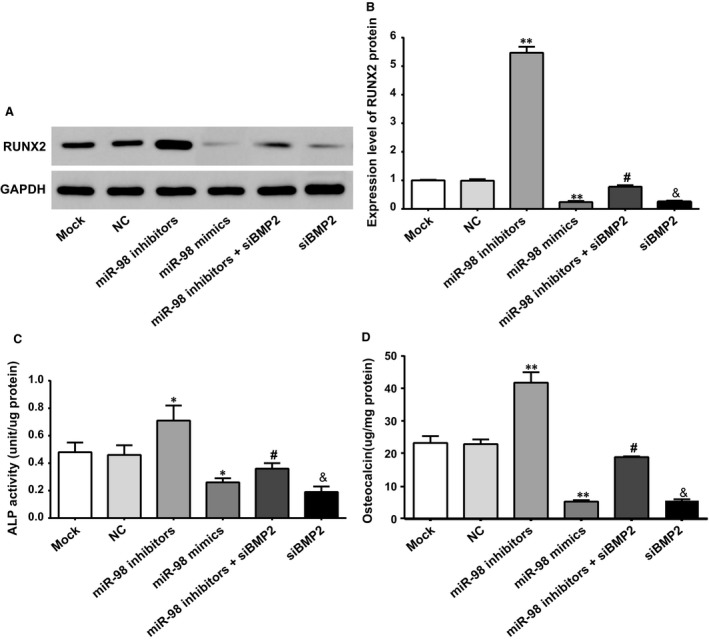
Levels of osteogenic markers in each group of cells. (**A**) The protein expression of RUNX2 in each group of cells; (**B**) ALP activity in each group of cells; (**C**) OC content in each group of cells; * represented the *P*‐value less than 0.05 compared with the Mock group and NC group; ** represented the *P*‐value less than 0.01 compared with the Mock group and NC group; # represented the *P*‐value less than 0.05 compared with the miR‐98 inhibitors group; & represented the *P*‐value less than 0.05 compared with the miR‐98 inhibitors + siBMP2 group. ALP: alkaline phosphatase; NC: negative control.

The miR‐98 mimics group showed a significantly decreased levels of RUNX2, ALP and OC compared to those in the NC group and the Mock group (all *P* < 0.05); the miR‐98 inhibitors group showed a significantly increased level of RUNX2, ALP and OC compared with those in the NC group and the Mock group (all *P* < 0.05); the NC group and Mock group showed no significant difference in the levels of RUNX2, ALP and OC (all *P* > 0.05). After osteogenic differentiation promoted by miR‐98 inhibitors, however, the levels of RUNX2, ALP and OC significantly decreased when transfected with siBMP2 (all *P* < 0.05). Compared with the miR‐98 inhibitors + siBMP2 group, the levels of RUNX2, ALP and OC significantly decreased in siBMP2 group (all *P* < 0.05) (Fig. 7A–C).

## Discussion

The hBMSCs samples used in our study were collected from patients undergoing hip arthroplasty. Density gradient centrifugation method was applied to obtain fresh hBMSCs, which consisted of mononuclear cells extraction and adherent culture. As for the morphological observation, the extracted and cultured hBMSCs were fusiform, fibroblast‐like cells, positive for mesenchymal cell surface molecules (markers like CD73, CD90 and CD105) and negative for hematopoietic stem cell related antigens (markers like CD14, CD20, CD34 and CD45), which were in accordance with the stem cell CD molecular phenotype. Consistent with our results, Zhang *et al*. found in their study that BMSCs were mainly spindle formed separate foci colonies and after 7–8 days culture proliferated at a fast speed 34. According to Choi *et al*., multipotent stromal cells showed spindle in shape and had a fibroblast phenotype 35. Commonly, various surroundings factors may influence the *in vitro* cultivation of hBMSCs, and different environmental factors may induce hBMSCs differentiated into osteoblasts, adipose cells, skeletal muscle cells, cartilage cells, smooth muscle cells or vascular endothelial cells 36, 37. Furthermore, BMP‐2 has been reported to play a leading role in the osteoblasts differentiation at an early stage 38. Thus, to better explore the effects of miR‐98 on osteogenic differentiation of hBMSCs by targeting BMP‐2, dexamethasone was added into the medium to induce the hBMSCs differentiation into osteoblasts 39. In our study, the osteogenic differentiation of the hBMSCs successfully induced, which was validated by staining method, indicating their functional specificity as stem cells. Therefore, the cells were verified to meet the requirements for further experiment.

In our study, we added EGF and PDGF into the cell culture medium to promote the hBMSCs proliferation and maintain the differentiation potential of hBMSCs. Epidermal growth factor and PDGF have been reported to be significantly enhanced the *in vitro* expansion capacity of MSC cultures 40, 41. Epidermal growth factor can enhance differentiation of MSCs and soluble EGF was proved to increase MSC proliferation, while preserves early progenitors within MSC population, which therefore cannot induce the cell differentiation 42. Further, the PDGF could regulate the wound healing and tissue regeneration by regulating the recruitment of fibroblasts and the precursor MSC and stimulating the generation of hydrogen peroxide which is required for cell migration 43, 44. It can be speculated that EGF and PDGF can be regarded as the safe mediums for MSC expansion and for clinical purposes 45, 46.

We searched for downstream target genes of miR‐98 with bioinformatics and found with dual luciferase reporter assay verification that BMP2 was one of the target genes of miR‐98 and the binding region of BMP2 gene 3′UTR and miR‐98 was highly conserved in mammals. The result echoed a previous report saying the predicted target sites of miR‐98 were located in regions that have high evolutionarily conservation 30. Transfection of miR‐98 mimics into hBMSCs led to downregulation of BMP2 mRNA and protein expression, RUNX2 protein expression, ALP activity and OC content, indicating that miR‐98 down‐regulated BMP2 expression at a post‐transcription level. We also found the osteogenic differentiation ability of hBMSCs was enhanced after down‐regulating miR‐98 expression; after transfection of siBMP2, the osteogenic differentiation inhibited by miR‐98 was weakened, which indicated the osteogenic differentiation inhibition of miR‐98 to hBMSCs by targeting BMP2.

BMPs belong to the transforming growth factor‐β (TGF‐β) family, including in mammals 33 members (TGF‐βs, nodal, activins and inhibins, myostatin and Müllerian‐inhibiting substance), and can be further divided into BMP‐2/4 group, BMP‐5/6/7/8 group (osteogenic protein‐1 group), growth and differentiation factor‐5/6/7 group and BMP‐9/10 group, of which most proteins of the BMP‐2/4 group induce formation of bone and cartilage tissues 47. BMPs bind to type I and II BMP receptors and signal through Smads and mitogen‐activated protein kinase pathways 48. BMP signalling is essential for chondrocyte differentiation *in vivo* and stimulates prechondrogenic cells differentiating into chondrocytes 49, 50. The growth factors involved in the formation and repair of bone and other connective tissues can be grouped into: insulin‐like growth factors, PDGF, fibroblast growth factors, EGF and proteins from the TGF‐β superfamily 51. The promotion of BMP2 on the osteogenic differentiation could therefore to some degree be granted. According to Hassan *et al*., miR‐23a played a negative role in osteogenic differentiation by suppressing runt‐related transcription factor 2 (Runx2), which was the key transcriptional factor to osteogenic differentiation 52. We then put forward a hypothesis that BMP2 may promoteosteogenic differentiation by up‐regulating Runx2.

Twenty miRNAs were identified to be consistently up‐regulated during osteogenic differentiation, of which miR‐10a, ‐22, ‐26a, ‐26b, and ‐29b recognized transcripts that encoded a set of proteins inhibiting osteogenesis 8. We have noticed the down‐regulated mRNA and protein expression of BMP2, which implied the effect of miR‐98 on target regulation of BMP2. This was validated by the surface markers of osteogenic differentiation, namely, the ALP activity and OC content. The specific mechanism of the targeted influence, however, needed to be verified in sufficient *in vivo* experiments. Although our results have confirmed that miR‐98 in hBMSCs could target the regulation of BMP2 expression, it was indicated that one miRNA could control multiple target genes, and one gene could be regulated by different miRNAs. Therefore, there might exist other related genes target‐regulated by miR‐98 in the occurrence and development of osteogenic differentiation of hBMSCs, which demanded further research.

In conclusion, we innovatively discussed the molecular mechanism of miR‐98 regulating the osteogenic differentiation of hBMSCs by targeting BMP2, specifically, knockdown of miR‐98 expression targeted to up‐regulate BMP2, whose role in promoting osteogenic differentiation of hBMSCs was then enhanced. We then believe that miR‐98 may act as a novel target for therapeutic application to promoting osteogenic differentiation. However, because of the limitations of our funds and time, the miR‐98 expression in different aged patients or different patients on the regulating the osteogenic differentiation of hBMSCs by targeting BMP2 was not tested. Hence, further studies with a wider range of aged participants are needed to be conducted to confirm the present results and underlying mechanisms.

## Conflict of interest

The authors have declared that no competing interests exist.
